# Structure of corneal layers, collagen fibrils, and proteoglycans of tree shrew cornea

**Published:** 2011-08-25

**Authors:** Turki Almubrad, Saeed Akhtar

**Affiliations:** Cornea Research Chair, Department of Optometry and Vision Sciences, College of Applied Medical Sciences, King Saud University, Riyadh, Saudi Arabia

## Abstract

**Purpose:**

The stroma is the major part of the cornea, in which collagen fibrils and proteoglycans are distributed uniformly. We describe the ultrastructure of corneal layers, collagen fibrils (CF), and proteoglycans (PGs) in the tree shrew cornea.

**Methods:**

Tree shrew corneas (5, 6, and 10 week old animals) and normal human corneas (24, 25, and 54 years old) were fixed in 2.5% glutaraldehyde containing cuprolinic blue in a sodium acetate buffer. The tissue was processed for electron microscopy. The ‘iTEM Olympus Soft Imaging Solutions GmbH’ program was used to measure the corneal layers, collagen fibril diameters and proteoglycan areas.

**Results:**

The tree shrew cornea consists of 5 layers: the epithelium, Bowman’s layer, stroma, Descemet’s membrane, and endothelium. The epithelium was composed of squamous cells, wing cells and basal cells. The Bowman’s layer was 5.5±1.0 µm thick and very similar to a normal human Bowman’s layer. The stroma was 258±7.00 µm thick and consisted of collagen fibril lamellae. The lamellae were interlaced with one another in the anterior stroma, but ran parallel to one another in the middle and posterior stroma. Collagen fibrils were decorated with proteoglycan filaments with an area size of 390 ±438 nm^2^. The collagen fibril had a minimum diameter of 39±4.25 nm. The interfibrillar spacing was 52.91±6.07 nm. Within the collagen fibrils, very small electron-dense particles were present.

**Conclusions:**

The structure of the tree shrew cornea is very similar to that of the normal human cornea. As is the case with the human cornea, the tree shrew cornea had a Bowman's layer, lamellar interlacing in the anterior stroma and electron-dense particles within the collagen fibrils. The similarities of the tree shrew cornea with the human cornea suggest that it could be a good structural model to use when studying changes in collagen fibrils and proteoglycans in non-genetic corneal diseases, such as ectasia caused after LASIK (laser-assisted in situ keratomileusis).

## Introduction

In recent years, the tree shrew has been used as a model for studying myopia [1,2]. Norton et al. [1] examined the effect of a period of continuous darkness on the refractive state and vitreous chamber depth of normal light-reared juvenile tree shrew eyes. They observed that four out of the five myopic eyes in the dark-recovery group became more myopic in darkness, and all the control eyes shifted toward myopia. The prevention of collagen cross linking and the expression of transforming growth factor-beta and collagen synthesis on the cornea and sclera of the tree shrew were also studied [3,4].

Ishiko et al. [5] observed the early ocular changes in a diabetic tree shrew model. They developed a tree shrew model of diabetes using streptozotocin (STZ) and studied the early ocular changes of diabetes (after one week of diabetes). Corneal autofluorescence was significantly increased one week after the STZ injection. These changes may be related to the impairment of the ocular homeostatic mechanisms due to the onset of diabetes. They suggested using the tree shrew as a model may make it possible to obtain diabetic ocular impairment data closer to human data than could be obtained in previous models of diabetes. Another advantage of using the tree shrew as a model is that tree shrews are not primates [6]. Given the ethical concerns surrounding the use of primates in medical research, the tree shrew could be used as an alternative to primates.

Few ultra-structural analysis studies have been performed on the tree shrew ocular system. McBrian et al. [7] used the tree shrew as a model to assess changes in the diameter of collagen fibrils in the sclera of myopic tree shrews. They reported a significant reduction in the median collagen fibril diameter at the posterior pole. Albon et al. [8] described the ultrastructure and immuno-histochemistry of the lamina cribrosa of the tree shrew. The authors showed the presence of multilayered connective tissue plates in the tree shrew lamina cribrosa LC stretched across the optic nerve canal at the level of the sclera and consisting of collagen types I, III, IV, V, and VI; and elastin and fibronectin. The ultrastructural distribution of collagen fibrils, elastic fibers, and axons has been shown by scanning and transmission electron microscopy. To our knowledge, there have been no studies performed on the ultrastructure of the tree shrew cornea.

A substantial proportion of the cornea consists of the stroma, which accounts for 95% of the corneal thickness in humans. The stroma is composed of parallel running lamellae which form the extracellular matrix. These lamellae are packed with uniformly distributed collagen fibrils of uniform diameters. This uniform distribution of collagen fibrils is responsible for the transparency of the cornea [9]. The uniform distribution of collagen fibrils is regulated by macromolecular glycoconjugates known as proteoglycans. The extracellular matrix of the cornea contains a class of small interstitial PGs known as collagen-binding small leucine-rich repeat proteoglycans (SLRRPs). These proteoglycans carry either kearatan sulfate chains or chondroitin sulfate⁄dermatan sulfate chains. Lumican, keratocan and mimican carry keratan sulfate chains, whereas decorin, biglycan and versican carry chondroitin sulfate⁄dermatan sulfate chains. Lumican is believed to be essential to the transparency of the cornea in that it regulates the uniform diameter of the collagen fibrils [10].

Ultrastructural features of the collagen fibrils and proteoglycans of the cornea have been discussed in various animal models, such as bovine, mouse, rabbit, chick, and zebrafish [10-13]. Detailed ultrastructural studies of Bowman’s layers and stroma in tadpoles, cattle, mice, rabbits, guinea pigs and humans were performed by Hayashi et al. [14]. This is the first paper to describe the ultrastructural organization of corneal layers, collagen fibrils and proteoglycans in tree shrew cornea.

## Methods

Tissue procurement and use was conducted in accordance with the Declaration of Helsinki and local regulations. It was ethically approved by the Local Ethical Committee of King Saud University, Saudi Arabia.

The corneal buttons of 5, 6, and 10 week old tree shrews and normal human corneas of 24, 25, and 54 year old males were fixed in 2.5% glutaraldehyde containing 0.05% cuprolinic blue (BDH Ltd, Dorset, UK) using a critical electrolyte concentration mode within 30 min of their removal from the eye of the dead animals [15]. The tissue was embedded in Spurr resin (TAAB Laboratories Equipment Ltd, Aldermaston, Berkshire, UK) and polymerised for 12 h at 70 °C. Three corneas were sampled from each age group, and five blocks from each cornea were used to cut cross-sections. Semi-thin and ultra-thin normal perpendicular cross-sections were cut with a Reichert-Jung Ultracut® microtome (Reichert-Jung, Vienna, Austria). The semi-thin sections were collected on glass slides and stained with toluedine blue. The sections were observed with an Olympus BX1 light microscope (Olympus, Tokyo Japan). Corneal layers were measured from the light micrographs taken by the light microscope. Ultra-thin sections were collected on 200 mesh copper grids. The sections were stained with uranyl acetate + lead citrate and phosphotungstic acid + uranyl acetate and observed by transmission electron microscopy (JEOL 1400; JEOL Ltd. Akishima, Japan). Digital images were captured with an Olympus 12 megapixel Qamisa camera using the ‘iTEM Olypmus Soft Imaging Solutions GmbH’ program (Münster, Germany). Five sections per cornea were observed using light and electron microscopy.

The minimum collagen fibril diameter and center-to-center spacing were measured using the ‘iTEM Olypmus Soft Imaging Solutions GmbH’ program. A decision was made to measure the minimum diameter of each fibril, rather than the average value to avoid errors due to any obliqueness in the fibril cross-sections. Akhtar et al. [15] measured the ‘minimum diameter’ of collagen fibrils of human cornea fixed in paraformaldehyde and embedded in LR White resin (The London Resin Co Ltd, Reading , Berkshire, UK). In the present studies, the ‘minimum diameter*’* of collagen fibrils of the cornea that were fixed in glutaradehyde plus cuprolinic blue and embedded in spurr resin was measured. The area size of the proteoglycans was measured in the anterior, middle and posterior stroma of the cornea. Data were analyzed using the statistic program SPSS (IBM Corporation, Armonk, NY). Kolmogorov–Smirnov and Mann–Whitney tests were performed. The mean and (pooled) standard deviation values for the 3 corneas were calculated taking into account the within-sample variances.

## Results

**Figure 1:**
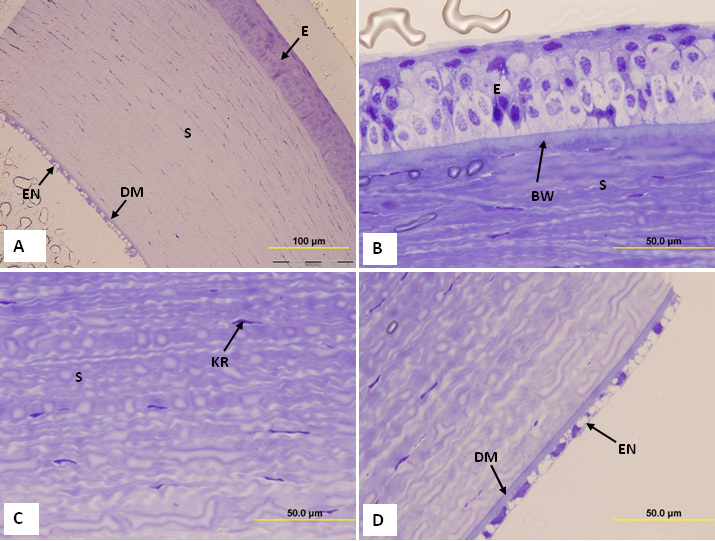
Light micrograph of tree shrew cornea. **A**: Tree shrew cornea consisting of epithelium, Bowman’s layer, stroma, Descemet’s membrane, and endothelium. Note the structure of the cornea is very similar to the human cornea and shows 5 layers. **B**: Part of the tree shrew cornea showing squamous cells, wing cells, basal epithelial cells and Bowman’s layer. **C**: Part of the corneal stroma containing keratocytes. **D**: Part of the cornea showing Descemet’s membrane and endothelium. B=Bowman’s layer, E=Epithelium, DM=Descemet’s membrane, KR=Keratocyte, EN=endothelium, and S=Stroma.

Light microscopic observations of semithin sections showed that the structure of the tree shrew cornea was very similar to that of the human cornea. The cornea was 320.96±2.95 µm thick and contained 5 layers: the epithelium, Bowman’s layer, stroma, Descemet’s membrane, and endothelium (Figure 1A-D). It was composed of squamous cells, wing cells and basal cells (Figure 1B). The stroma consisted of collagen fibril lamellae containing keratocytes (Figure 1C). The posterior part of the stroma was covered by Descemet’s membrane and a single layer of the endothelium (Figure 1D).

Light microscopic measurements of corneal layers from semi-thin sections of tree shrew and human cornea fixed in glutaraldehyde are shown in Table 1. The epithelium of the tree shrew cornea was 50.29±1.62 µm thick and constituted 15% of the corneal thickness (Table 1). The thickness of the Bowman’s layer and stroma of the tree shrew were 5.53±1.0 µm and 258±7.0 µm, respectively. The tree shrew Bowman's layer constituted 2.13% and the stroma constituted 80% of the entire cornea (Table 1). In the human cornea, the epithelium was 50±2.00 µm thick, the Bowman's layer was 13±0.58 µm thick, and the stroma was 554±10.39 µm thick. In the human cornea, the epithelium, Bowman's layer and stroma constituted 8%, 2.34%, and 88% of the thickness of the cornea, respectively (Table 1). All the data presented in this paper in relation to the human cornea were produced during the course of the present study.

**Table 1:** Measurement of the corneal layers of tree shrew and human cornea fixed in cuprolinic blue in 2.5% glutarldehyde and embedded in spur resin.

**Corneal layers**	**Tree shrew center CuB staining embedded in Spurs resin (µm)**	**Human center CuB staining and embedded in Spurs resin (µm)**
Cornea	320.96±2.95	629±7.21
Epithelium	50±1.62	50±1.58
Bowman’s layer	5.53±1.0	13±0.58
Stroma	258±7.00	554±10.39
Descemet’s membrane	3.24±0.02	11±1
Bowman's layer % of cornea	1.7%	2.06
Strom % of the cornea	80%	88%
Epithelium percentage	15.6%	8%
Bowman’s layer (BW) % of the stroma	2.13% of stroma	2.34% of stroma

**Figure 2:**
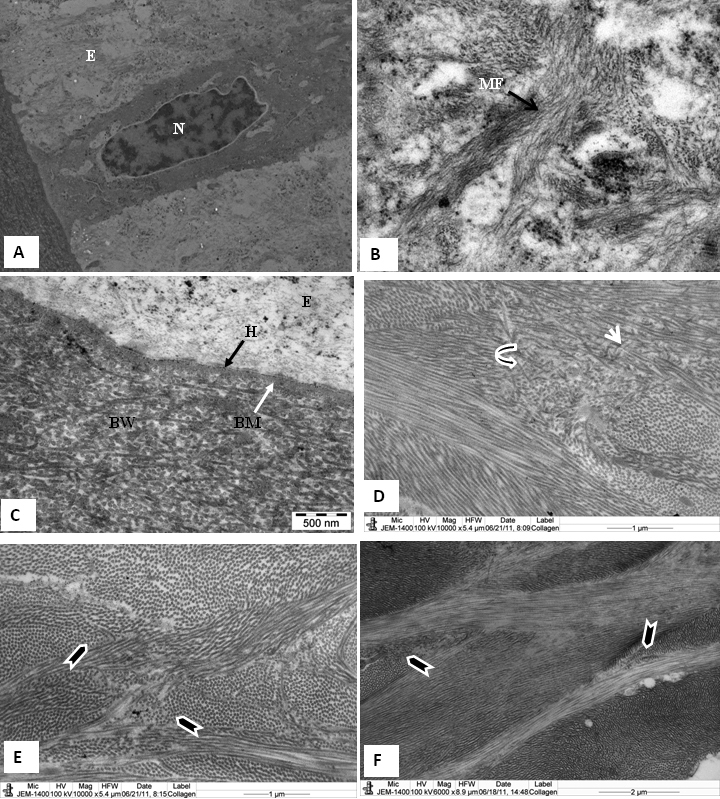
Electron micrograph of tree shrew and human cornea fixed in 2.5% glutaraldehyde containing cuprolinic blue and embedded in spurr resin. **A**: Basal epithelial cells are columnar and contain large nuclei. **B**: Prominent cytoplasmic filaments in basal epithelial cells. **C**: Basal epithelial cells attached by hemidesmosomes to a basement membrane followed by a Bowman’s layer consisting of dense, randomly arranged collagen fibrils. **D**: Non linear, random distribution of collagen fibrils (curved arrowhead) present in the anterior stroma just below the Bowman's layer. Some of the collagen run across the longitudinally-running collagen fibrils (arrowhead). **E**: Lamellae are interlacing (arrowhead) in the anterior stroma of the tree shrew. **F**: Lamellae are interlacing (arrowhead) in the anterior stroma of the normal human cornea. B=Bowman’s layer, BM=Basement membrane, CF=Collagen fibrils, E=Epithelium, H=Hemidesmosomes, and MF=Cytoplasmic filaments.

Ultrastructural observations showed that the basal epithelial cells were columnar and contained large nuclei and prominent cytoplasmic filaments (Figure 2A,B). They were attached to a basal lamina by hemidesmosomes (Figure 2C). The Bowman’s layer consisted of dense, randomly arranged collagen fibrils similar to those of the human cornea (Figure 2C).

**Figure 3:**
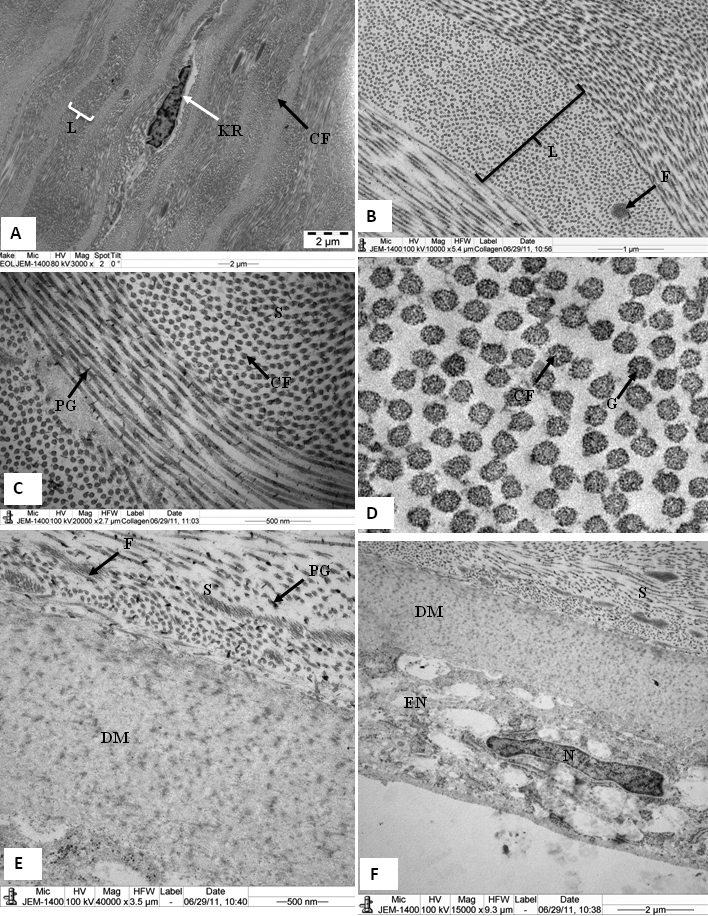
Electron micrograph of tree shrew cornea fixed in 2.5% glutaraldehyde containing cuprolinic blue and embedded in spurr resin. **A**: In the middle stroma, parallel running lamellae containing a keratocyte. **B** and **C**: Lamella containing orderly, packed collagen fibrils and proteoglycans. **D**: In cross-section, collagen fibrils exhibiting tiny particles, some of which are of high electron density. **E**: Pre-Descemet's stroma containing very fine fibrils and large PGs around the collagen fibrils. Fibrillar structures are present throughout the Descemet's membrane. **F**: Part of the posterior cornea, showing a banded Descemet's membrane and an endothelium containing a nucleus; also a prominent endoplasmic reticulum. CF=Collagen fibrils, DM=Descemet's membrane, EN=endothelium, F=Fine fibril, G=tiny particles, KR=Keratocytes, L=Lamella, PG=Proteoglycan, and S=Stroma.

The major component of the cornea was the stroma, which consisted of lamellae. In the anterior part of the stroma, the lamellae interlaced antero-posteriorly (Figure 2D,E). There was a nonlinear, random distribution of collagen fibrils (curved arrowhead) present in the anterior stroma, just below the Bowman's layer (Figure 2D). Some of the collagen fibrils ran across the longitudinally-running collagen fibrils (arrowhead; Figure 2D). The lamellar interlacing (arrowhead) in the anterior stroma of tree shrews (Figure 2E) were very similar to lamellar interlacing (arrowhead) in anterior stroma of human cornea (Figure 2F). In the middle and posterior stroma, the lamellae ran in the plane of the cornea, with neighboring lamellae crossing at varying angles (Figure 3A). Keratocytes were present in between these lamellae (Figure 3A). Within each stromal lamella, the collagen fibrils were packed in an orderly, parallel array (Figure 3B,C). These collagen fibrils were decorated with proteoglycans (Figure 3B,C). In cross-section, the collagen fibrils exhibited tiny particles, some of which were of high electron density (Figure 3D). The Descemet’s membrane showed only a banded region (Figure 3E,F). In the pre-Descemet's stroma, proteoglycans were very large in size (1,525 nm^2^; Figure 3E). There were numerous very thin microfibrils present in the stroma (Figure 3B) and in the pre-Descemet's area (Figure 3E). The endothelial cells contained a large nucleus and all the normal cell organelles (Figure 3F).

**Figure 4:**
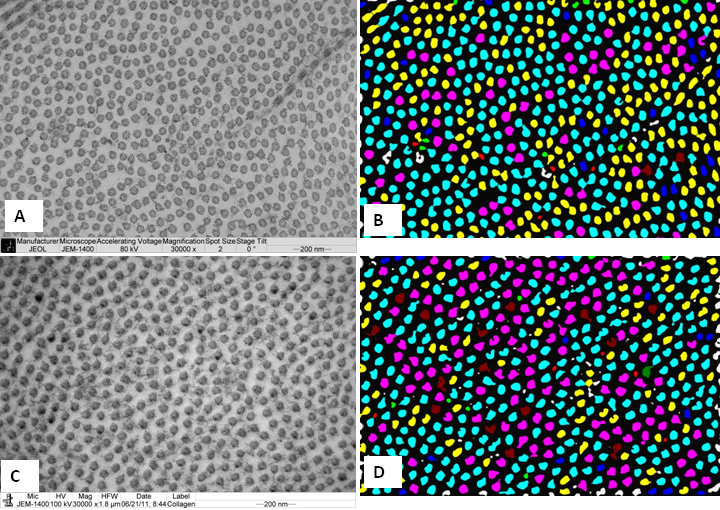
Electron micrograph of tree shrew and human cornea fixed in 2.5% glutaraldehhyde containing cuprolinic blue and embedded in spurr resin. **A**: Electron micrograph of collagen fibrils of tree shrew cornea. **B**: Digital image obtained after processing the image shown in **A**. **C**: Electron micrograph of collagen fibrils of human cornea. **D**: Digital image obtained after processing the image shown in **C**. The images were displayed by using color coding to demonstrate the distribution of the variable diameters of collagen fibrils. Collagen fibril color coding: Red=15–20 nm, Green=20–25 nm, Blue=25–30 nm, Yellow=30–35 nm, Aqua=35–40 nm, Pink=40–45 nm, and Brown=45–50 nm.

The ‘minimum diameter’ of the collagen fibrils and the 'proteoglycans mean area' of the cornea were analyzed by digital image analysis (Figure 4A-F). Electron micrographs of the collagen fibrils of the tree shrew (Figure 4A) and human cornea (Figure 4C) were color coded according to the size of their diameters (Figure 4B,D). The digital images of the tree shrew (Figure 4B) and human cornea (Figure 4D) contained collagen fibril diameter ranges of 20 nm-50 nm. The ‘minimum diameter’ in the tree shrew cornea was 39±4.25 nm, and in the human cornea it was 38.65±4.37 nm (Table 2). The interfibrillar spacing in both the tree shrew and human cornea were 52.91±6.07 nm and 52.52±5.36 nm, respectively (Table 2).

**Table 2:** Measurement of collagen fibrils, interfibrillar spacing, and proteoglycans mean area of tree shrew and human cornea fixed in cuprolinic blue in 2.5% glutarldehyde and embedded in spur resin.

**Animals**	**Diameter minimum (nm)**	**Interfibrillar spacing (nm)**	**Proteoglycan mean area (nm^2^)**
Tree shrew cornea	39.00±4.25	52.91±6.07	390±438*
Human cornea	38.65±4.37	52.52±5.36	93±74

*Statistically significant difference with p<0.05.

**Figure 5:**
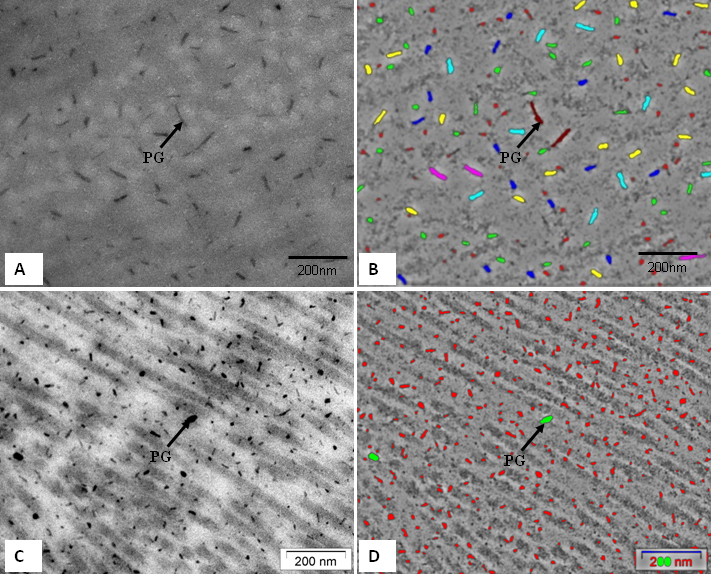
Electron micrograph of tree shrew and human cornea fixed in 2.5% glutaraldehhyde containing cuprolinic blue and embedded in spurr resin. **A**: Electron micrograph of proteoglycans of tree shrew stroma. **B**: Digital image obtained after processing image shown in **A**, showing variable area distribution of PGs. **C**: Electron micrograph of proteoglycans of human stroma. **D**: Digital image obtained after processing image shown in **C**, showing variable area distribution of PGs. PG=Proteoglycan; Proteoglycans color coding: Red=50–350 nm^2^, Green=350–650 nm^2^, Blue=650–950 nm^2^, Yellow=950–1,250 nm^2^, Aqua=1,250–1,550 nm^2^, Pink=1,550–1,850 nm^2^, and Brown=1,850–2,150 nm^2^.

Electron micrographs of the proteoglycans (PGs) of the tree shrew stroma (Figure 5A) were color coded according to the size of the PGs, as shown in the digital image (Figure 5B). The PGs’ area was calculated from digital images. The mean PG area of the tree shrew cornea was 390±438 nm^2^. The largest PGs area in the middle stroma was approximately 2,131 nm^2^. The mean PG area of the pre-Descemet's region was 1,525 nm^2^. The process of digitizing the electron micrographs, as outlined for the tree shrew above, was followed for the proteoglycans of the human stroma (Figure 5C). The digitized images are shown in Figure 5D. The mean PG area of human cornea was 93±75 nm^2^. The PGs of tree shrew stroma were significantly larger than those of human cornea (Table 2).

## Discussion

The normal human cornea consists of five layers, namely the epithelium, Bowman’s layer, stroma, Descemet’s membrane, and endothelium. Similar to the human cornea, the tree shrew cornea also has five layers. The thickness of the epithelium of the tree shrew cornea was similar to that of the human cornea, but the tree shrew Bowman's layer and stroma were thinner than those of the human cornea. In the tree shrew, the epithelium constitutes 15% of the corneal thickness, whereas the stroma constitutes 80% of the corneal thickness. This is very similar to the human cornea, in which the epithelium constitutes approximately 8% of the corneal thickness and the stroma constitutes 88%. The tree shrew cornea also lacked a non-banded zone in the Descemet's membrane. The human postnatal cornea has a non-banded zone, which increases in thickness over the life span. The absence of such a zone in the tree shrew corneas studied may relate to the young age of the specimens. Tree shrews in captivity may live for up to 12 years of age. The overall thickness of tree shrew cornea was approximately half that of the human cornea. In spite of these differences, the ratios between the Bowman's layers and the entire cornea in both tree shrews and humans were similar. The tree shrew Bowman's layer constitutes 2.13% of the cornea, and human Bowman's layer constitutes 2.34% of the cornea.

The structure of tree shrew cornea is very different from that of other animals, such as the murine, bovine, and rabbit. Of particular interest and differing from that of the murine or the bovine cornea is the occurrence of a Bowman’s layer. Hayashi et al. [14] have reported the thickness of Bowman's layer (BW) in relation to the stromas of various animals’ corneas fixed in glutaraldehyde plus osmium tetroxide and embedded on TAAB epoxy resin. According to the authors, the percentages of BW thickness in relation to the stromal thickness was very small in mice (1.4%), rats (1.2%), rabbits (1.2%), cattle (0.6%), and human (3.2%). Our studies showed that the percentage of BW in relation to the stroma in tree shrews and humans are 1.8% and 1.9%, respectively. This suggests that the structures of the tree shrew Bowman's layer and stroma are very similar to human BW and stroma. Although other animals do have BWs, they are not similar to the human BW. In addition to the tree shrew, a Bowman’s layer is also found in primate, avian, and some zebrafish corneas [13,16,17]. Please note there is a difference in the percentage of the BW layer thickness (3.2%) of human cornea fixed in osmium plus embedded in TAAB epoxy [14] and the BW layer thickness (1.9%) of the human cornea fixed in cuprolinic blue plus spurr resin in our studies. This suggests that the fixation method and resin affect the thickness of the BW.

Another interesting similarity between the tree shrew cornea and the human cornea is the organization of the stroma into an anterior woven zone and a posterior zone of parallel lamellae. In addition to this, the high magnification images produced in this study showed the presence of electron-dense particles within the collagen fibril cross-sections, which were very similar to the particles observed within the collagen fibrils of the human cornea [15]. It is presumed that these particles are either proteoglycans or pro-collagen fibrils within the collagen fibrils.

An analysis of the collagen fibrils suggested that the density of the collagen fibrils/µ^2^, interfibrillar spacing and ‘minimum diameter’ of tree shrew cornea were similar to those of human cornea. There was no significant difference between the collagen fibril diameter of tree shrew and that of human cornea. It must be emphasized that, in previous studies [15], the ‘minimum diameter’ of the collagen fibrils was calculated from human cornea processed in 4% para-formaldehyde and embedded in LR White resin. The diameter was 24 nm in LR white embedded tissue. In the present studies, calculations of the ‘minimum diameter’ of the collagen fibrils were taken from cornea processed in glutaraldehyde containing cuprolinic blue dye and embedded in spurr resin. The ‘minimum diameter’ of cornea embedded in spurr resin was 39±4.25 nm. There is a significant difference between the two diameters of the collagen fibrils processed using the two methods. Further studies are required to analyze the variation in collagen fibril diameters when the cornea processed by different methods. In spite of the similarities in collagen fibril organization, there were significant variations in the PGs’ mean area of the tree shrew and human cornea. The PGs’ mean area of tree shrew (390 nm^2^) was significantly higher than the PGs’ mean area (90 nm^2^) of human cornea reported by Akhtar et al. [15]. The PGs’ mean area in the pre-Descemet's region was also very high (1,525 nm^2^).

Recently, various animal models, such as rats, mice, cattle, chicks, and zebra fish have been used to study corneal complications. Chicks have been used as a model to study wound healing, subepithelial keratomileusis (LASEK) and the effect of hyaluronic acid on the cornea [18-20]. The distribution of the collagen fibrils and proteoglycans were studied in the cornea of cattle, rats, chicks, and zebra fish [10,13]. The mouse has also been used as a diabetic model to analyze the changes in collagen and proteoglycans in diabetic conditions [21]. In recent years, the zebra fish has been considered as a candidate model to investigate the thinning and increased size of the scleral coat [22].

The similarities between the tree shrew cornea and the human cornea described above suggest that the tree shrew will be a better model as compared to other animal models when used to study the causes of the thinning of the cornea, as well as changes in collagen fibrils and proteoglycans in pathological conditions, such as diabetes. The occurrence of a Bowman’s layer similar to that in humans also suggests that the tree shrew will be a good model to assess swelling and its effect on the collagen fibrils and proteoglycans. The tree shrew cornea could also be a good model to study the collagen fibrils and proteoglycans present with non-genetic diseases, such as ectasia caused after LASIK.
